# Engaging with Film to Teach Students About Virtuous Outsourcing of Cognitive Tasks to LLMs

**DOI:** 10.1007/s44206-025-00234-2

**Published:** 2025-11-14

**Authors:** William Gopal, Michael Quinn

**Affiliations:** University of Glasgow, 67 Oakfield Avenue, Glasgow, G12 8LP Scotland

**Keywords:** Philosophy of education, Virtue epistemology, Intellectual virtue, Film, AI, Cognitive offloading

## Abstract

Recently there has been a call to deploy AI technologies in secondary education. An accompanying worry is that if students became overly reliant on LLMs, they would become cognitively deskilled - and this would be an impediment to the growth of their cognitive character. One pedagogical approach aiming to offset this worry consists of teaching students how to critically evaluate the outputs of LLMs while they are using them – the Use-First Critical Thinking approach (UFCT). We argue that UFCT alone cannot assuage this worry insofar as it misses an important, prior step in equipping students with the skills to be aware of, and reflect upon, by what lights decisions whether to delegate tasks to LLMs are appropriate. We propose an alternative pedagogical approach involving film, specifically *Memento*, which allows students to identify delegative choice points and what (in)appropriate delegation looks like.

## Introduction

The marked increase in the technical sophistication and availability of AI technologies has led to calls for their deployment in various sectors of society, including primary and secondary education. We use the term “EdAI” to refer to the deployment of AI technologies in primary and secondary education. Proposals surrounding EdAI include teacher-use cases (e.g., using AI systems to generate lesson plans); administrator-use cases (e.g., using predictive algorithms to identify students likely to fail or drop out); and student-use cases. Specifically, one student-use case is to use large-language-models (LLMs), such as ChatGPT, within the classroom to generate practice problems, provide explanations of content, retrieve information, or act as a conversational partner (Kasneci et al., 2023; Wieczorek et al., 2024).

This proposal is accompanied by a concern that introducing LLMs into the classroom would lead to the cognitive deskilling of students, such as the diminishment of critical thinking skills (Kasneci et al., 2023) or reducing independent thought (Akgun & Greenhow, 2022) if students become overly reliant, in the short term or long term, on LLMs to complete tasks.^1^ To bring this worry into focus, if students become overly reliant on using LLMs to provide digestible summaries of a primary text, rather than reading the text itself, students’ abilities of reading comprehension might diminish because they are no longer cognitively active. In other words, in being overly reliant on an LLM students no longer flex their cognitive muscles, leading them to (potentially) atrophy. Spelling the worry out further, if cognitive deskilling in the form of a diminishment of critical thinking were to occur, then students are at risk of being misled or forming false beliefs based on accepting an LLM’s output.^2^

One way of offsetting the worries associated with this specific student-use case is to shift teaching practices and develop pedagogies for students to learn (i) how to use (and in some cases even design or build) LLMs and (ii) what the appropriate use (and in some cases design) of LLMs in educational contexts would be (Rowe, 2019; Bartoletti, 2022). Current pedagogical proposals (see e.g., Mollick and Mollick (2023a, b) developed along these lines consists of what we classify as use-first critical thinking (UFCT) approaches:

*Use-First Critical Thinking* (UFCT)

Students are taught how to write good prompts for a variety of LLM use cases via prompting LLMs (i.e., *using* them) and evaluating the outputs using critical thinking skills.

We take the central idea of UFCT to be as follows. Through prompt writing, output evaluation, and prompt evaluation students remain cognitively active by using and developing cognitive skills associated with writing prompts and, importantly, using skills associated with critical thinking during the evaluation process. Since students remain cognitively active on these grounds, worries of cognitive deskilling are assuaged.

However, as the worry about cognitive deskilling goes, merely introducing LLMs in the classroom risks fostering overreliance upon LLMs and thereby the deskilling of students. As such, what are the grounds to think that the most fitting pedagogical approach available is one in which students are guided to think critically about the quality of outputs *whilst using* LLMs? Furthermore, even if such an approach is appropriate to the extent students are capable of proficiently using LLMs, does it equip students with the tools to identify how the mere decision to use an LLM might amount to being epistemically irresponsible?

In the *pars destruens* of this paper, we argue that UFCT misses an important, *prior* step – namely, that students are not taught to identify in what cases it is appropriate to use LLMs to perform or aid in completing intellectual labour. Our concern is that UFCT incorrectly assumes that pedagogical approaches should involve activities in which students are explicitly guided to think critically about the quality of outputs *whilst using* LLMs. In other words, UFCT is analogous to students learning to drive before learning the highway code; whilst one would be able to control the car effectively, one would lack an awareness of the rules and norms which govern how to navigate certain interactions such that they may place oneself, and others, in harm’s way. In the *pars construens*, we provide a pedagogical approach which functions as a broad corrective to deskilling through LLM use. More specifically, we propose using excerpts from Christopher Nolan’s film *Memento* (2000) to develop students’ awareness of, and reflections on, certain intellectual virtues related to the appropriate use of LLMs.

Here’s the plan. In Sect. 2, we describe and critique Use-First, Critical Thinking (UFCT) as a pedagogical approach which has students critically engage with LLMs while they are using them. In Sect. 3, we spell out the reasoning behind the worry that students would be cognitively deskilled if LLMs are introduced into the classroom, showing that this is not a new concern brought about by LLMs, but rather an instance of Pritchard’s (2016) technology-education tension. Then, we identify the requirements a pedagogical approach must meet to assuage the worry of cognitive deskilling, arguing that UFCT fails to do so. Keeping these requirements in mind, we propose a teaching task (Teaching Task 1) based on *Memento*. In Sect. 4, we argue that unless students’ assumptions surrounding LLMs are challenged, the worry of cognitive deskilling has greater weight. Then, we provide Teaching Task 2 in which students create a handbook, again based on *Memento*, to scaffold the challenging of such assumptions. In Sect. 5, we identify the upshots of our approach, highlight the implications of our critique of UFCT and contextualise our findings in the wider picture of contemporary AI use in education.

## Use-First, Critical Thinking (UFCT)

We take the aim of UFCT approaches as equipping students with the skill of thinking critically about the outputs of LLMs. This is achieved by pedagogical activities where students *use* LLMs *whilst* learning how to evaluate their outputs using skills of critical thinking. To see UFCT in action, consider Mollick & Mollick’s (2023a, 11–18) activity where students could learn how to appropriately use an LLM as a personal tutor (e.g., providing new information related to preestablished learning goals). They suggest students write different prompts for the LLM whilst the teacher provides guidance about which parameters of the prompt to change, and then, whilst under the guidance of a teacher, apply evaluative criteria to the outputs of the LLM.

Whilst the guidance in Mollick & Mollick’s (2023a;2023b) proposal is not developed as fully as we may wish, we assume that, with enough development, UFCT succeeds in showing students how to evaluate the outputs of an LLM by honing their critical thinking skills. Our contention, however, is that UFCT is not concerned with encouraging students to consider whether they should use an LLM to perform intellectual labour in the first place. For example, suppose a student, Annie, is revising for her finals but struggling to understand a topic. She could either ask ChatGPT to write her a draft of an answer to the exam question or persevere and attempt to work things out for herself. Suppose Annie chose the former, she might miss a formative experience in becoming intellectually perseverant and be worse off for it. That is, even if Annie does not err by forming false beliefs or being misled when using ChatGPT, she might nonetheless err in the sense that by using ChatGPT, she sacrifices a chance of improving her cognitive character.

Whilst this kind of opportunity loss is pervasive (e.g., focusing on one’s science coursework sacrifices the opportunity to revise for a history exam), our concern isn’t the lost opportunity *after* making the decision, but rather a lost opportunity *whilst* making the decision. Our worry is that making decisions about whether to delegate intellectual labour to LLMs are opportunities to improve one’s cognitive character in their own right. However, if such delegative choice points remain unrecognised as opportunities of this kind, students will continually miss them. Furthermore, this missed opportunity is not one which could simply be rectified elsewhere or at a different time, since it occurs from Annie’s failure to consider, perhaps notice, the choice of whether to use the LLM in any given case. The key point is that the completion of cognitive tasks, whether aided or unaided by artefacts, is essential to the development of one’s cognitive character. Such tasks include deciding whether to delegate intellectual labour to LLMs (or other artefacts). However, this task of deciding whether to delegate intellectual labour to LLMs (or other artefacts) cannot itself be delegated (see: Carter, 2019; Turner, 2022) but must be performed “manually” (i.e., unaided).^3^ In this paper, we are primarily concerned with this group of cognitive tasks. Crucially, if students fail to undertake these tasks, such as by failing to notice a delegative choice point, then students will continually miss out on opportunities to develop their cognitive character.

UFCT, whether by chance or design, does not and cannot prepare students for the following: first, to be aware of when they’re at a delegative choice point (i.e., the decision whether to outsource a task to an LLM); second, to begin to reflect on navigating delegative choice points well – both of which we think are essential. Just as Annie’s error comes from a lack of awareness and reflection surrounding her delegative choice point, the same can be said for UFCT as a pedagogical approach. That is, UFCT does not cater for awareness nor reflection on deciding when to use LLMs. Even if UFCT were to remain neutral on improving students’ awareness of deciding when to delegate, this continues to place them at risk of cognitive deskilling. We return to this worry in Sect. 3.2. Having provided a reconstruction of UFCT and identified our concerns, we now turn to how the deskilling worry relates to the importance of students considering how intellectual labour should be delegated.

## The Deskilling Worry, Delegative Intellectual Labour, and Virtue

Whilst worries regarding LLMs cognitively deskilling students are prominent, the underlying concern that using technologies within educational settings might negatively impact students’ cognitive abilities and skills is nothing new, nor necessarily linked to the technical sophistication of technologies such as LLMs. Pritchard (2016) identifies such worries as arising from a tension between a view about the purported aims of education and how, upon a specific picture of cognition, technology-use is understood to conflict with these aims – call this the technology-education-tension.

To establish this tension, Pritchard (2016) takes an aim of education to be the cultivation and improvement of students’ cognitive abilities and skills in a way that improves students’ cognitive character such that they can engage in real-world epistemic challenges.^4^ To grasp the idea, consider the cognitive processes one undertakes to complete a weekly grocery shop in accordance with a budget. We’ve got to calculate whether buying a multi-pack of toilet roll is not only cost-efficient but feasible given our budget. A role of education then, in this specific case, is to prepare students to successfully perform these sums, that is, to cultivate and develop the skills and abilities associated with mental arithmetic.

Such skills and abilities are essential in navigating a variety of situations we encounter within our cognitive lives, and crucially, we can perform these cognitive tasks in better, or worse, ways. For example, we might do our mental arithmetic too hastily, or carefully; further still, one might be *overly* cautious and triple-check the sums. In this respect, the way we habitually undertake and perform certain cognitive tasks develops and expresses our cognitive character (e.g., being intellectually perseverant or open-minded). These character traits are intellectual virtues in the Aristotelian sense (for clarity, we use cognitive character (traits) and intellectual virtue interchangeably).^5^ The aim, then, in this aspect of educating, is to not only ensure that it equips students with the cognitive abilities and skills required for epistemic success (like those in the example above) but also to develop students’ cognitive character such that they are *virtuous* knowers i.e., by allowing them to learn, practice, habituate, and master their cognitive abilities and skills expressive of a virtuous cognitive character.^6^

However, note that in the example above, the cognitive abilities and skills described are performed *internally* within one’s head (i.e., intracranially). What if these cognitive abilities and skills could be performed externally by using a calculator or a budgeting app? Likewise, what if, at school, one never learned how to do the mental arithmetic required but only how to use a calculator? According to Pritchard (2016) herein lies the technology-education tension. If the improvement of students’ cognitive agency or intellectual virtue (i.e., their cognitive character) is cultivated only through exercising their *intracranial* cognitive abilities and skills but technologies can be used to *extra-cranially* perform these skills/abilities, doing so would not contribute to the development of students’ cognitive character. We take the worry that students would be cognitively deskilled if LLMs are introduced into the classroom (Kasneci et al., 2023; Akgun & Greenhow, 2022) to be an instance of the technology-education-tension (Pritchard, 2016) as has been described here.

According to Pritchard (2016), this tension can be dissolved if we shift the picture of cognition as being something that occurs only within the head, to one in which cognitive processes also occur *beyond* the skull. That is, if the use of external artefacts can support, replace, or constitute cognition and education also cultivates these cognitive abilities and skills, then education can develop students’ cognitive character. Whilst Pritchard (2016) focuses on *extended* cognition, we focus primarily on embedded and enactive cognition as, given our focus on LLMs, we do not advance and defend the stronger claim that students’ use of and possible reliance upon LLMs will meet the conditions proposed for cognitive extension (for these conditions see: Carter & Kallestrup, 2020; Clark, 2008; Heersmink, 2015; Menary, 2012; Pritchard, 2010).^7^

Instead, we take using LLMs within education to be instances in which students can cognitively offload tasks and focus on how education might cultivate the cognitive skills, abilities, and character traits associated with responsible cognitive offloading. Cognitive offloading (Risko & Gilbert, 2016) occurs when an agent delegates a cognitive task to an artefact such that the completion of the cognitive task is realised or supported by the artefact. For example, one might offload the cognitive task of calculating the most cost-efficient size of toilet roll to a calculator or partially offload the task of navigation by using Google Maps; or, in the context of education, a student might cognitively offload the task of gathering information about how to write a well-structured exam answer by using ChatGPT. With this in mind, we can now express the reasoning behind the worry that students would be cognitively deskilled if LLMs are introduced in the classroom (Kasneci et al., 2023; Akgun & Greenhow, 2022).

### Two Conditions for Offsetting the Deskilling Worry

Given the breadth of purported use cases of LLMs within education, if LLMs are implemented within education, students are likely to often encounter decisions whether to cognitively offload some, or all, intellectual labour to an LLM. And, if students decide to cognitively offload intellectual labour to an LLM too frequently, then they will no longer perform (nor habituate and cultivate) cognitive abilities and skills such that their cognitive character won’t improve.^8^ From this, we can express the concern that students will be cognitively deskilled through LLM use as follows.

deskilling worry

If LLMs are implemented in education, then students will no longer perform (and thereby habituate and cultivate) cognitive abilities and skills such that their cognitive character will not improve.

We dissolve deskilling worry by identifying the types of cognitive task/intellectual labour which cannot be offloaded to an artefact and then argue that a necessary condition for an educational approach which can quell deskilling worry is that it equips students with the skills associated with completing these types of intellectual labour. This is because students have no choice other than to perform the cognitive abilities and skills required for completing these tasks and, as such, can continue to develop their cognitive character.

According to Carter (2019), one type of intellectual labour which cannot be offloaded are the cognitive tasks required for using and managing technologies such that we can offload to them. We call this higher-order intellectual labour. To see why, consider a student attempting to offload the task of gathering information to an LLM. For a student to succeed in this, they must know how to use LLMs in a way that makes them a useful cognitive aid, for example, how to design and input prompts such that the required information is made available to them in the LLMs’ output. That is, the cognitive skills and abilities required for using an artefact are a pre-requisite for offloading intellectual labour to an artefact and, as such, cannot be offloaded to an artefact.^9^

A second type of intellectual labour which cannot be offloaded to artefacts is delegative intellectual labour; that is, the intellectual labour associated with *deciding* whether to offload a cognitive task to an artefact (Carter, 2024, p. 134). To see this idea in action, let’s return to the example of completing a grocery shop and calculating the most cost-efficient pack of toilet-roll; here, one is faced with a choice to either perform the task of calculation unaided or offload it to a calculator. Likewise, recall Annie – the student revising for her finals but struggling to understand a topic. She could look to exemplary past papers and content provided by her teacher and work things out for herself, or she could prompt an LLM to provide a draft of an exam answer. Choice points like these require one to evaluate whether one should cognitively offload – we take it that this process of evaluation (partially) constitutes delegative intellectual labour.

Pulling things together, if, following the introduction of LLMs into education, students are likely to often face decisions whether to cognitively offload to LLMs, then students might make appropriate or inappropriate decisions to offload intellectual labour to LLMs (i.e., excessively or scarcely).^10^ If they decide to offload excessively, then they might not improve their cognitive character because they no longer perform certain cognitive tasks and thereby do not habituate or cultivate certain valuable cognitive abilities and skills. However, if students decide to offload appropriately, then it’s plausible that their cognitive character might still improve insofar as there will be certain cognitive skills and abilities to cultivate which are associated with delegative intellectual labour. So, if LLMs are introduced into education, students might either offload to LLMs excessively such that their cognitive character doesn’t improve, or they offload appropriately such that their cognitive character can still improve.

If it’s not the case that students are taught the skills and abilities of delegative intellectual labour, then students are more likely to offload to LLMs excessively and thereby not improve their cognitive character, whereas if students are taught the skills and abilities of delegative intellectual labour, they will be more likely to offload appropriately and can still improve their cognitive character.^11^ Therefore, if LLMs are introduced into education for students to offload appropriately and be able to improve their cognitive character, then students must be taught the skills and abilities associated with delegative intellectual labour.

Our position, then, is that the worries of LLMs cognitively deskilling students can begin to be offset only if students learn the skills associated with delegative intellectual labour *and* higher-order intellectual labour. That is, offsetting deskilling worry can begin only if students are taught how to use and manage LLMs *and* how to identify when they ought (not) offload intellectual labour to LLMs. To this end, we argue that a pedagogical approach in which students reflect on specific case scenarios of when and when not to delegate certain tasks, highlighting the possibility that deskilling could occur through their unchecked LLM use. Encouraging such reflection will then, we believe, better prepare students to deploy such reflections when it comes to further specific cases. In what follows, we argue that whilst UFCT teaches students skills associated with higher-order intellectual labour, it does not teach those associated with delegative intellectual labour, thus failing as a pedagogical proposal to offset deskilling worry.

### UFCT Does Not Address Delegative Intellectual Labour

Recall that higher-order intellectual labour pertains to the cognitive tasks associated with using and managing technologies such that we can offload certain cognitive tasks (Carter, 2019, p. 15). Here, a further distinction might be drawn between (i) higher-order intellectual labour *enabling* cognitive offloading (enabling-labour) i.e., the cognitive skills and abilities required to offload and (ii) that which *improves* the process of offloading (improvement-labour). To bring this into relief, consider the difference between learning how to use iCalendar such that one can input their upcoming schedule in comparison to learning how to use keyboard shortcuts to set up shared calendars or re-occurring events. The former type of intellectual labour is that which enables somebody to cognitively offload the task of remembering one’s schedule to iCalendar, whereas the latter type is related to managing iCalendar so they can offload more effectively.

Given that part of UFCT is educating students how to write prompts, and assuming UFCT is successful in this respect, then UFCT equips students with the skills and abilities required to cognitively offload tasks to LLMs. In this respect, UFCT provides an opportunity for students to engage in *enabling-*labour pertaining to using LLMs. How does the approach fare for improvement-labour, that is, with how to manage LLMs more effectively?

For Mollick & Mollick (2023a; 2023b), UFCT consists in educating students how to write *good* prompts for a variety of use-cases (e.g., LLM-as-tutor, LLM-as-partner in “think-pair-share” discussions) via using LLMs and *then* evaluating the outputs of differing exemplar prompts. The good-making features of prompts are ones in which possible pedagogical risks and general risks associated with LLMs are offset. One such pedagogical risk being that the LLM may respond to students’ requests to directly provide answers, as opposed to engaging in collaborative problem-solving if LLMs are used as tutors (Mollick & Mollick, 2023a, pp. 8–9). To offset such a pedagogical risk, students are provided with examples of prompts (e.g., “How does this example relate to the concept under discussion? ) and a brief explanation of *why* prompts are worded in this way (e.g., to provide sufficient contextual detail). Assuming that UFCT is successful in its aims in cases like these, UFCT does provide an opportunity for students to engage in *improvement-*labour. As such, UFCT equips students with the skills associated with higher-order intellectual labour.

However, recall that a pedagogical approach can be successful in offsetting deskilling worry only if students learn the skills associated with higher-order intellectual labour *and* delegative intellectual labour. Our contention is that UFCT does not equip students with the skills associated with delegative intellectual labour due to its pedagogical requirement for students to *use* LLMs. If UFCT is to be implemented, students will use LLMs and as a result they will have already decided to delegate some intellectual labour to an LLM (i.e. the teaching tasks of UFCT). And if this is the case, then *ex hypothesi*, UFCT cannot offer guidance to students about how to develop skills associated with delegative intellectual labour (i.e., evaluating whether delegation is (in)appropriate). Whilst Mollick and Mollick (2023a, p.5) suggest students must have the choice to opt-out of AI assignments, their approach does not teach students *why* they might want to opt-out. That is, their approach does not offer students explanatory reasons why not to use LLMs from which they could evaluate their decision. Here, a proponent of UFCT might suggest that their approach be accompanied by an explanation as to *why* they might want to opt-out by outlining the risks associated with AI use to students. However, even if UFCT provides such explanatory reasons, it assumes that students can evaluate the decision about whether one should delegate intellectual labour to an LLM based on the information provided. That is, UFCT is not concerned with having students identify what good delegative decisions look like *and* the skills to make good delegative decisions. As such, for UFCT to equip students with the skills associated with delegative intellectual labour, a prior step is required – namely, educating students to be able to *identify* what a good delegative decision is.

However, the defender of UFCT might reply that such a response implies an overly rigid picture of the application of critical thinking skills.^12^ Here’s the line of argument. Granting that students possess a general ability to think critically about whether they should undertake some course of action, they are in a good position to apply this to other, novel, considerations – and in our case specifically, whether to opt-out of AI assignments and whether to delegate intellectual labour to LLMs. That is, if UFCT provides explanatory reasons as to why they might want to opt-out, students do, in fact, have the necessary skills to think critically and decide whether to opt out.

For example, just as a student might decide to opt-out of a sports class because it would be beneficial to instead spend that time revising for an upcoming exam, they might also decide to opt out of AI assignments because of, say, the risks associated with the propensity of LLMs to output false or biased information. After being presented with these risks students might exercise their critical thinking skills to decide whether to delegate intellectual labour to an LLM because, say, the expediency of using an LLM outweighs the possible drawbacks. So, it’s not the case that UFCT fails to offer guidance about how to develop skills associated with delegative intellectual labour viz. evaluating whether delegation is (in)appropriate. Rather, UFCT does offer guidance in the form of being a novel case to apply, and further hone, pre-existing critical thinking skills.

Here, we concede that students will, and do, in fact have this general ability and it is applicable to the decision to opt out. However, that students might decide to opt-out of AI assignments or abstain from delegation based on exercising a general ability to think critically doesn’t entail that this decision is made for the right kinds of reasons. That is, even if the decision to opt-out of AI assignments or abstain from delegation is made by exercising one’s critical thinking skills, this decision can be made without evaluating the decision by the lights of the normative standards that establish what counts as a good (or bad) delegative decision. In other words, it can be made on the absence of considering a normative reason. As it stands, UFCT does not provide students with the guidance to understand, or consider, such reasons i.e., those which determine whether a delegative decision is good by the lights of norms governing delegative decisions. This is the gap our proposal fills insofar as it provides pedagogical tasks enabling students to distinguish and identify good delegative decisions from bad delegative decisions (see Sect. 3.3). To do this let’s first briefly consider what a *good* delegative decision is.

According to Carter (2024, p.144), looking to the norms of intellectual ethics (viz., those which govern how one ought to take up certain inquiries and how long to pursue them) provides some initial guidance about when (not) to delegate. He argues that those who comply with the norms of intellectual ethics possess virtues of inquiry (e.g., open-mindedness, intellectual perseverance) which guide decisions about what questions one takes up and how and when to settle them (e.g., through trusting another’s testimony, or investigating for oneself). In the context of offloading virtues of inquiry, these virtues might also aid in deciding when to engage or abstain from delegation.^13^ Specifically, Carter (2024, p.142–145) argues that the intellectual virtues regulating good delegative decisions will involve, at least, the following two dispositions – namely: to be sensitive to non-epistemic values (e.g., practical, ethical, and political) by which some inquiries are considered more worthwhile than others – call this *sensitivity*; and to reliably assess the limits of one’s ability to judge whether the decision to delegate would place oneself in a position to know – call this *reliable placement*.^14^

Taking stock, we have argued that UFCT misses an important preliminary step, viz., enabling students to identify what a *good* delegative decision looks like (i.e., those exhibiting *sensitivity* and *reliable placement*). For students to be able to identify this, they would benefit from being able to answer the following questions:

Q1: What intellectual virtues should be exercised when an agent is deciding whether to offload intellectual labour to a cognitive artefact?

Q2: What intellectual virtues should be exercised when an agent is deciding whether to offload intellectual labour to an LLM?

We now turn to providing a pedagogical approach in which students engage with the film *Memento*, tasked with identifying whether the delegative decisions made by Leonard, the main protagonist, exhibit *sensitivity* and *reliable placement* as per Q1.

Our pedagogical proposal introduces students to philosophical concepts through *Memento*, specifically those required for an awareness of when they’re at a delegative choice point and to begin reflecting on navigating this decision. Importantly, we are not making the stronger claim that our proposed approach aims to, nor can, equip students with the dispositions of *sensitivity* or *reliable placement.* While students would undoubtedly be able to learn about which virtues are associated with good delegative decisions, our approach is neither necessary nor sufficient for the development of these virtues.^15^ Rather, we suggest that a pedagogical approach which involves introducing philosophical concepts by engaging with selected excerpts from a film (in our case *Memento*) to make the decision whether or not to delegate to LLMs salient to students and eventually to have them reflect on such questions from a virtue theoretic perspective. Within this wider context, students will be better able to understand and evaluate the importance of intellectual virtue as it relates to using LLMs in the classroom. In this sense, we suggest engaging with selected parts of *Memento* to teach specific aspects related to cognitive offloading.^16^ If this pedagogical approach involving film can achieve our specified pedagogical goals concerning LLM use, then engaging with *Memento* becomes attractive.

Why, though, *Memento* specifically? Before answering this question, we describe the core aspects of the film. In *Memento* (Nolan, 2000) Leonard Shelby, a former insurance investigator, seeks revenge for the murder of Catherine, his wife. Leonard develops anterograde amnesia as a result of an injury sustained during Catherine’s murder. To compensate for his inability to create new memories, and to organise facts and information about Catherine’s killer, Leonard develops a ‘system’. This system includes annotated polaroids, notes, tattoos and rules governing when and how he inscribes information he hopes to remember. However, as the film progresses, the viewer learns that Leonard’s system is fatally flawed. It transpires that Leonard avenged Catherine’s murder some time before the events of the film, and he has been leading himself down rabbit holes by manipulating his own memory system. Teddy, the crooked cop who claims to have been helping Leonard, confesses that he used Leonard to take down criminals by misleading him and channelling his anger.

*Memento* explores several issues related to cognitive offloading in a visceral way which we believe students will find compelling. Whilst *Memento* is a complex film with an ambiguous ending, the way in which we suggest engaging with the film bypasses worries related to students having to wade through these complexities. Indeed, we suggest that the film not even be watched in full by the class to avoid getting lost in interpretations. Rather, we argue that it is important for students to see how Leonard’s (flawed) system works, which itself is illustrative of cognitive offloading. In being shown relevant clips from the film, students will be able to evaluate Leonard’s actions of cognitive offloading viz. the creation and use of his system. Later, they will be directed to consider their findings within the context of cognitive offloading and LLMs.

We also believe that there are more general but significant pedagogical upshots to engaging with *Memento* in the context of having students consider the appropriate use of LLMs, namely: (i) interactive engagement by engaging with selected examples of visual media, (ii) an understanding of the intellectual virtues associated with good delegative decisions, and (iii) the application of (i) and (ii) to identify how LLMs might be used to cognitively offload responsibly. We return to these upshots in Sect. 4, as in the next two sections we are focused on how engaging with *Memento* addresses our worries with the UFCT approach.

### Teaching Task 1

The aim of Teaching Task 1 is to introduce students to the concepts of cognitive offloading and delegative intellectual labour which serve as necessary precursors for responsible AI use in which deskilling is avoided. To do this, the manner in which Leonard cognitively offloads would be directly explored early on and would be framed as ‘Leonard’s system’ (Quinn, forthcoming). Throughout this task, the focus would be on students becoming aware of what it is to cognitively offload to a given artefact. Subsequently, Teaching Task 2 will reflect on what, if any, impact this would have on guiding students towards considering deskilling through LLM use and associated risks.

After watching selected excerpts of *Memento*, with teachers drawing attention to salient scenes, students, placed in groups, would be asked what practical problems they would face in their life if they had a condition like Leonard’s and their answers compared and contrasted. They would then be asked what did and did not work with Leonard’s system. For example, how would they improve his system, or would they instead just develop their own from scratch? What would that look like? Why would they choose to either improve Leonard’s system or develop their own? What would count as an improvement? Answering these questions would involve thinking deeply and critically about how Leonard builds his system, how his system works, and evaluating the systems’ efficacy. In this opening task, students would be considering in what ways Leonard offloads tasks to his system. When students go on to evaluate Leonard’s system, they will be encouraged to critically analyse answers to the question of whether cognition occurs only intracranially or whether these can be supported, or perhaps constituted (see Quinn, forthcoming), one’s interactions with external artefacts. Subsequent tasks could begin to draw comparisons between Leonard’s system of offloading pieces of information to various artefacts (e.g., Post-it notes, annotated polaroids) to remember them and his intracranial system of memory. This evaluative part of the task raises students’ awareness about cognitive offloading, and in comparing Leonard’s system with intracranial memory, students are being guided towards considering what tasks can be performed by an artefact, rather than by the subject themselves.

After this evaluative task is completed, a group discussion about what evaluative standards students have adopted will be facilitated by the teacher, addressing content relevant for starting to identify whether Leonard’s actions satisfy Carter’s (2024) picture of good delegative decisions. The purpose of this section of the task is to introduce students to the notion of intellectual virtue and guide them in grounding their evaluation of Leonard’s delegative decisions. To achieve this, teachers might group together student responses as to why they think that Leonard’s system is effective or flawed. Here, student responses might range from agreeing that Leonard is, for example, intellectually perseverant in a similar manner to the exemplar, or disagree and claim that Leonard is instead intransigent.

Following this, and depending on their answers, students would be asked to discuss how Leonard might become more, or less, virtuous in his actions. For example, students who answer that Leonard is intellectually perseverant would be asked to consider ways Leonard might act habitually such that he is no longer perseverant but intransigent or irresolute. Alternatively, students who answer that Leonard is intransigent or irresolute would be asked to consider ways Leonard might begin to habitually act such that he is perseverant. Later, after students have discussed whether Leonard’s actions are virtuous, students will be asked to compare and contrast, first individually then as a group, their answers to an exemplar who satisfies *sensitivity*.

To aid in enabling students to identify when Leonard faces delegative choice points, and eventually whether the decisions are good, the final portion of this task aims to introduce students to the distinction between higher-order intellectual labour and delegative intellectual labour. First, students would be presented with a selection of scenes from *Memento* in which Leonard is undertaking either higher-order intellectual labour (e.g., Leonard’s dialogue explaining how his practices support and provide the foundations of a broader system) or delegative intellectual labour (e.g., Leonard’s explanation of his decision to get only key pieces of information tattooed on his body).

Second, students would be asked what the differences are between Leonard’s intellectual actions are in these scenes. This could be set up as a broad group discussion, thereby allowing the teacher to facilitate and guide students in identifying that Leonard is, in one case, gathering evidence, whereas in the other, he is managing his cognitive artefacts. Then, building on their answers to the comparative task about virtue, students would be asked if there were any dispositions Leonard would need to have, due to his condition and reliance on his system, which another exemplar, who does not have Leonard’s condition, would not need. This could be set-up as a group task in which students are provided with a list of character traits that include accessible descriptions of each disposition and then have students sort them into categories “Leonard”, “Exemplar”, “Both”. Once again, students would be comparing the completion of certain tasks by the subject alone with when the subject uses an artefact to delegate in order to draw out the important distinctions between these two cases.

We believe that Teaching Task 1 addresses some of the worries we have addressed in the context of UFCT. By its very nature, Teaching Task 1 addresses the concern that UFCT does not offer students time and space to consider delegative decisions regarding LLM use. In Teaching Task 1, students are deliberately given time and space to consider whether they should use an artefact *before* using it. Note, however, that we are not yet asking students to consider the possible consequences of delegating (i.e., deskilling). Rather, we are merely drawing the comparison of completing cognitive tasks with or without an artefact. In doing so, we are raising student awareness about what it means to delegate such tasks before asking them what the consequences might be (which we do in Teaching Task 2).

Relatedly, the framing of ‘Leonard’s system’ encourages students to consider what dispositions should be exercised (which is made easier by identifying Leonard’s conspicuous lack of these dispositions), and to further reflect on whether they should engage with certain artefacts in the first place. As such, students are encouraged to become more sensitive to how non-epistemic values play a role in regulating what good delegative decisions are, as identified by Carter (2024). Students therefore become increasingly aware of the non-epistemic value of, say, practicality as they are guided towards considering how effective Leonard’s system is in terms his use of particular artefacts (such as a polaroid or a tattoo). They do this by inquiring into the specific differences between completing a task alone compared with an artefact. In assessing the differences here, students will be in a better position to move onto considering *reliable placement*. We demonstrate this progression in Teaching Task 2 which is more focused on reflection rather than awareness.

At this stage, the completion of Teaching Task 1 ensures that students are made aware of what it means to cognitively offload and the ways they offload tasks in their daily lives. *Memento*, in offering a visceral depiction of offloading, gives students the opportunity to offer Leonard advice on how to manage his system efficiently. Furthermore, in offering Leonard advice, students may consider in what ways their advice may apply to them individually, setting the stage for the reflective exercises in Teaching Task 2.

Taking stock, we have argued that to offset worries about the introduction of LLMs into education leading to the cognitive deskilling of students, education must equip students with the necessary skills associated with delegative intellectual labour *and* higher-order intellectual labour. However, we identified that UFCT presupposes that students possess the skills to identify what good delegative decisions look like. As a result, we suggested engaging with *Memento* to encourage students to consider norms underpinning delegation. In what follows, we raise a second worry about UFCT: that the approach fails to challenge students’ assumptions about LLMs, or at worst potentially reinforce them, such that students might inappropriately offload intellectual labour to LLMs.

## Virtue, Delegation, and LLMs

The underlying concern beneath deskilling worry – viz. that, when done excessively, cognitive offloading atrophies our cognitive abilities – does not only emerge from technologies becoming increasingly sophisticated; compare, for example, Socrates’ comment in the *Phaedrus* (275) on how the invention of writing would produce forgetfulness as people would no longer exercise their memory. However, given the “hype” surrounding LLMs and AI systems (e.g., how companies producing LLMs and EdAI often market such applications as panaceas and a force for overwhelming good with very few drawbacks) it’s likely that students will possess strong opinions about the possible capabilities, benefits or drawbacks of LLMs within the classroom.^17^ As such, we have purposefully opted to cast Q1 without specifying whether the agent is offloading to an LLM or a cognitive artefact more generally. This grants students more time to reflect on what is at stake when offloading to cognitive artefacts, before considering LLMs specifically.

Given this, we want students to approach Q2 in such a way that they are primed to be critical of the idea that an artefact being used for cognitive offloading is somehow indexed to an artefact’s technological sophistication. If students already know that cognitive processes can be offloaded to analogue artefacts, such as pen and paper, polaroids, or Post-it notes, and of associated epistemological problems (as achieved by the completion of Teaching Task 1), then students might approach cases involving LLMs in a more systematic and principled manner. This is because students will be aware that delegative decision points subject to the norms of intellectual ethics emerge in the use of less technically sophisticated artefacts (e.g., pen & paper) *as well as* more sophisticated artefacts such as LLMs. That is, students are focused on reflecting whether a delegative decision would be appropriate rather than misfocusing on the technology.

The broader hope, then, is that students will be aware that some of the virtue epistemic problems (viz. excessive offloading inhibiting the development of one’s cognitive character)^18^ related to LLMs and EdAI, are not wholly limited to, or brought about by, the presence of AI systems in the classroom and elsewhere. Rather, they will begin to consider that some of the virtue epistemic problems related to LLMs and EdAI are much wider reaching. For example, they may reflect on problematic LLM use outside of the classroom and how it can bring about cognitive deskilling regardless of where it is used, and that deskilling is likely to occur if any given agent consistently delegates tasks to LLMs without constraint. If students were to possess a wider awareness of LLMs and EdAI, it is reasonable to think that they may be more sensitive to the inflated claims from EdAI companies about the benefits of using LLMs within education as well as beyond. With this more cautious backdrop informing their opinion of LLM use in education and elsewhere, students will be better primed to consider the act of delegating itself as an important task. Furthermore, if students possess an awareness of the virtue epistemic issues related to less technologically sophisticated artefacts, this might plausibly help them draw distinctions between what, exactly, is different or similar in cases involving LLMs. This will then allow students to identify whether an artefact’s technological sophistication may contribute to a difference in virtue epistemic issues. Irrespective of whether this is the case, they will nonetheless be carefully reflecting on the implications of interacting with artefacts, and by virtue of considering artefacts through a more critical lens, we argue, students are more likely to interact with such artefacts appropriately.

### Teaching Task 2

Teaching Task 2 further develops students’ responses to questions about which intellectual virtues are required in making decisions whether to offload to cognitive artefacts in order to avoid deskilling. As per Q2, in this teaching task students will explore what intellectual virtues should be exercised when deciding whether to offload intellectual labour to an LLM. The task requires students to identify and assess Leonard’s cognitive character throughout his journey, and then apply this to the use of LLMs. To achieve this, we suggest that students, throughout the unit, create a ‘Philosopher’s AI Handbook for Leonard Shelby’. The handbook, structured as a series of questions, scenarios, and dilemmas serving as prompts for student responses, consists of two thought-experiments in which Leonard encounters an LLM.

Similarly to Teaching Task 1, students will become aware of the importance of considering whether it is appropriate to use an LLM, especially when such use might lead to being cognitively deskilled. However, students will also be encouraged to reflect on such decisions, with the virtues regulating good delegative decisions. Having already considered *sensitivity*, this task addresses *reliable placement* and elicits a more conspicuous awareness and reflection on whether a delegative decision contains a reliable assessment of the limits of one’s ability to judge whether the decision places oneself in a position to know.

The first thought experiment consists of Leonard building an LLM – *LeonardGPT*. Students are presented with a case where Leonard digitises all his notes which serves as the training data for *LeonardGPT*. To explore the importance of this, a compare and contrast task could be set up in which students compare the similarities and differences between how Leonard’s use of analogue cognitive artefacts and *LeonardGPT* might place him in a position to know. Then, students would be asked to evaluate if Leonard attempted to assess whether his decision to delegate places him in a position to know and whether this assessment is limited by his abilities. Here, students will build from the more general considerations of offloading they developed in Teaching Task 1 and, importantly for our purposes, will become aware that one can also cognitively offload to LLMs. This exploratory thought experiment sets the scene for having students consider the importance of how delegative labour may or may not place one in a position to know.

The second thought experiment aims at introducing problems of dependency on LLMs. This thought experiment aims to get students to consider the lack of control Leonard has over completing tasks in the absence of an LLM. This is achieved by having students creatively explore a thought experiment in which the digitised notes Leonard uses to create *LeonardGPT* are destroyed, and Leonard does not, due to his condition, remember or know the information he used to create *LeonardGPT*. When attempting to complete a task, such as deducing Teddy’s motives, Leonard discovers that *LeonardGPT* no longer works, and that he can only complete selected cognitive tasks (such as arithmetic, comprehending text and writing) because of his dependency on the LLM. Students would be tasked with writing a short piece of creative writing that analyses how he came to be in this situation. Of course, our task is guiding students towards reflecting on Leonard’s deskilling as a result of being overly dependent on the LLM, and we would want them to consider the notion that Leonard did not, in this instance, reliably assess the limitations of his ability to judge whether his delegative decisions placed him in a position to know.

As a result, the writing piece would not be from the perspective of Leonard, but rather from a third person narrator. Students would then be able to analyse the situation in a manner unavailable to Leonard, given his condition. Here, Leonard’s inability to create new memories would serve as the catalyst for the task because he would evidently be confused at the unusual situation he was in. Successful completion of the task would depend on students focusing on Leonard’s problematic delegation of tasks and tracing his current predicament back to the way he engaged with *LeonardGPT*. Namely, students would explore whether Leonard properly considered how the development and use of *LeonardGPT* would impact whether he’s in a position to know. To be clear, Leonard’s epistemic capabilities are challenged by the manner of his delegation of tasks *and* his memory problems, but by setting up the task to focus on the former (for instance through questions which ask students how Leonard’s *decisions* have led to the problems he now faces), students will be engaged in evaluating the nature of Leonard’s deskilling.

In having students evaluate Leonard’s deskilling, this task encourages engagement with Q2; specifically, it aims to develop students’ awareness that excessive LLM use is directly related to deskilling and can be partially avoided through engaging in delegative choices. It also has students begin to reflect on *reliable placement* as they are assessing the limits of Leonard’s ability to judge whether the decision to delegate would place himself in a position to know. Given that Leonard’s decision to delegate led him to a position where he was unable to complete certain tasks, and to develop certain skills, students could therefore assess in what ways Leonard’s excessive offloading also resulted in him not being able to collect and retrieve information in the way that he did before using *LeonardGPT.*

These thought experiments deliberately position students to reflect on the importance of considering whether to delegate intellectual labour to LLMs, with a specific focus on the importance of appropriate use in relation to deskilling. Importantly, student evaluations would consist of an active and imaginative exploration of the complex intricacies involved with using LLMs and are closely linked with examples from the film. By the end of Teaching Task 2, students would have a substantial notebook written from the perspective of Leonard progressing through both variations of the *LeonardGPT* thought experiments. In so doing, students have opportunities to reflect on when they are at a delegative choice point, what the potential consequences of excessive delegation are, and the importance of navigating these choices points in line with *sensitivity* and *reliable placement*.

Whilst providing a comprehensive description of assessing these tasks is beyond the scope of this paper, we now turn to identifying the minimal success criteria for students completing these teaching tasks. Students complete the handbook to a satisfactory level if and only if they have developed an *awareness* of what virtues are associated with good delegative decisions with regards to LLMs. To do this, a substantive response to both teaching tasks, consisting of a demonstration of their ability to use examples from the film to support their ideas and to think creatively about how Leonard would act in these new situations, would be required. By demonstrating a critical awareness of the implications of Leonard’s interactions with cognitive artefacts more broadly, and *LeonardGPT* specifically, responding to these teaching tasks requires students to develop and exercise the skills of identifying what the drawbacks of excessively using LLMs are, and how they might begin to counter them effectively. That is, they identify the importance of making good delegative decisions.

Note that this identification is exactly what the UFCT approach cannot address. Recall from Sect. 3.1- Sect. 3.2, that two necessary conditions had to be met to offset deskilling worry, namely that in their education, students are equipped with skills associated with (i) delegative intellectual labour and (ii) higher-order intellectual labour. Whilst UFCT satisfies the latter condition, it does not satisfy the former. This is because UFCT requires an additional, prior step viz., the consideration of whether one should delegate to LLMs in the first place. Whilst our approach satisfies the former condition, it does not satisfy the latter. Given that our pedagogical approach is compatible with students being taught how to critically think about the outputs of LLMs *after* they have completed the teaching tasks we have proposed, a combination of our approach and UFCT is well positioned to meet the conditions required to offset deskilling worry. Though to be clear, we have not made the stronger claim that our approach will be sufficient in offsetting deskilling worry. Likewise, note that the approach we have offered here equips students with the skills to *identify* what good delegative decisions look like (viz., those guided by Carter’s (2024) virtues); we are not proposing an approach which equips students with the skills to *make* good delegative decisions nor develop such virtues.

As before, we identify how Teaching Task 2 (TT2) further addresses the worries which were acknowledged in Teaching Task 1 (TT1). Like TT1, TT2 also, by its very nature, offers time and space for students to consider using LLMs given that it takes place *before* engaging with LLMs directly. However, whereas TT1 had students reflect on the relevance of intellectual virtues when engaging with artefacts more generally, TT2 is more specifically geared towards intellectual virtues and LLM use. By having students engage in exploratory thought experiments, based on clips from *Memento*, they are able to conceptually explore how the intellectual virtues they considered more generally in TT1 now apply to the specific use of LLMs in TT2. By having students consider Leonard’s predicament, they are tasked with reflecting on whether delegative decisions are good or bad insofar as they exhibit *sensitivity* or *reliable placement*. As we noted, UFCT is unable to meet this condition because by having students use LLMs while they critically evaluate their outputs, they are unable to consider whether their decision to offload to an LLM is good or bad, by the norms of intellectual ethics.

Similarly to TT1, using Leonard as an exemplar is effective insofar as it is a compelling demonstration of when someone does not consider intellectual virtues when cognitively offloading. Building on this in TT2, students will therefore be primed to consider what effects this perspective would have on LLMs more specifically. Therefore, students will be able to reflect specifically on salient concerns related to responsible LLM use. We have argued that UFCT is simply not capable of having students consider such concerns, particularly in the context of cognitive deskilling, since its focus remains on the quality of outputs rather than identifying what counts as a good or bad delegative decision.

### Upshots

Having described the success criteria for the Teaching Tasks and our motivations behind them, we now identify three more general but significant upshots of our approach for students, teachers, and educators. These upshots are as follows: (i) interactive engagement with selected examples of visual media,^19^ (ii) an understanding of the intellectual virtues associated with good delegative decisions, and (iii) the application of (i) and (ii) to identify how LLMs might be used to cognitively offload responsibly.

As we have demonstrated, engaging with *Memento* to learn about intellectual virtues can encourage sustained and improved engagement of students. Engaging with clips from *Memento* provides a clear and effective scaffold and, rather than explaining the concepts directly, they can use clips from the film to help assist in teaching them. Finally, for educators more widely, this approach addresses a welcome pedagogical shift toward developing interdisciplinary skills necessary for responsible use of LLMs. Given the current prevalence of ChatGPT, discussions of this nature are already taking place and engaging with a fictional narrative to scaffold the learning of cognitive offloading and virtues associated with good delegative decisions offers both students and teachers the space and confidence to discuss their ideas. Also, given that LLMs are already being widely used in a variety of contexts, it is important that students see how it is applicable to various aspects of their lives. Studying how intellectual virtue relates to LLMs through visual media encourages an interdisciplinary approach which brings together different aspects of the curriculum such as film, literature, philosophy, and ICT. We maintain that a unified approach for learning about EdAI offers a secure platform for educators to develop meaningful learning experiences for students.

With our pedagogical approach, students will have an understanding of what intellectual virtues are associated with good cognitive offloading. This, in turn, will empower them in their use and interactions with LLMs and will also be applicable for other forms of technology. As for teachers, our approach encourages further opportunities to create engaging lesson units relating to intellectual virtues as well as for wider philosophical ideas. Regarding educators more widely, engaging with clips from *Memento* to learn about responsible LLM use offers up to date pedagogical tasks in which the importance of responsibly using LLMs is brought to the fore given how LLMs are becoming more mainstream in day-to-day life and in education.

With the previous two upshots in mind, students will be able to apply these benefits when using LLMs. Engaging with *Memento* also has the added benefit of allowing students to identify and evaluate delegative choices in a low-stakes environment as they are forming conclusions about a fictional character rather than expressing personal judgements on others. Finally, in the context of wider curricula aims, our approach signals a shift away from “use-first, critical thinking” approach, thereby allowing students to engage in the conversation about the wider implications of LLM use in educational contexts and outwith.

## Conclusion

We have argued that, as a pedagogical approach, UFCT does not equip students with the cognitive skills and abilities pertaining to delegative intellectual labour (viz., evaluating when delegating/not is appropriate) and as a result UFCT fails to meet a necessary condition for offsetting the worry that introducing LLMs into education would inhibit the development of students’ cognitive character. Then, we provided a pedagogical approach which fills this lacuna. In engaging with *Memento* conceptually, students will be better positioned to offset potential cognitive deskilling when using LLMs as they will develop a more thorough understanding of what it means to delegate tasks to an LLM and the potential pitfalls of offloading too frequently.

Considering and identifying intellectual virtues, we maintain, is essential for appropriate LLM use which UFCT has been shown to be incapable of addressing. In arguing for the importance of students reflecting critically on the use of LLMs, with reference to cognitively offloading, we have suggested a pedagogical approach by engaging with clips from the film *Memento.* In contrast to UFCT approaches, we have argued that our proposal of using Leonard’s system as a conceptual exploration will benefit students in considering the various complexities of considering when it is appropriate to use LLMs. We maintain that engaging with selected clips from the film is effective for scaffolding the apprehension of complex philosophical concepts, to help visualise cognitive offloading, and that our proposed teaching tasks are ways in which teachers could explore, via *Memento* excerpts, the intellectual virtues associated with virtuous LLM use.

## Data Availability

We do not analyse or generate any datasets, because our work proceeds within a theoretical approach. Data sharing not applicable.

## Footnotes

1 Note that the concern about cognitive deskilling is not necessarily linked to the use of LLMs within education: see Pritchard (2016); Turner (2022). For a broader discussion of deskilling and technology and an argument that there are some skills conducive to one’s wellbeing which over reliance on technology poses a risk to, see Fricker (2021).

2 For reason to think that users of LLMs might need to further scrutinise the outputs of LLMs, as opposed to say the contents of a textbook or the testimony of another human, consider the fact that LLMs are prone to output “hallucinations”. That is, to output false or misleading information presented in a confident and plausible tone. Note that even if no cognitive deskilling takes place, there are independent reasons to think that students, and users of LLMs more broadly, are at risk of forming false beliefs on the basis of the output of an LLM, and as a result may need to critically evaluate such outputs. For discussion of whether we are epistemically justified in believing on the basis of an output of an AI system see: Carter (2024, 125–134); Freiman (2024;2023); Durán and Jongsma (2021); Sullivan (2022).

3 For an overview on the relation between the development of one’s cognitive character and flourishing (and virtue theoretic epistemology more broadly), see Kelp (2020). To be clear, we are not claiming that *only unaided* completion of cognitive tasks are conducive to the development of one’s cognitive character. For an argument that completing cognitive tasks aided by artefacts contribute to the development of one’s cognitive character see Battaly (2018). Rather our claim is that the completion of a specific subset of cognitive tasks conducive to the development of one’s cognitive character cannot be delegated to LLMs (or other artefacts) – namely the cognitive task of deciding whether to delegate other cognitive tasks to an artefact.

4 Here, and throughout, we follow Pritchard (2016) in taking cognitive abilities and skills (i.e., acquired abilities) to be reliable belief-forming processes.

5 For more on character based intellectual virtues, see Zagzebski (1996), Baehr (2011), Sosa (2015).

6 For more on intellectual virtue and education see papers in Baehr (2016); see also Kotzee et al. (2021).

7 Here, we remain neutral as to whether the use of LLMs would qualify as being cognitive extensions. For an account of how cognitive extension may obtain with ChatGPT specifically in educational settings see: Cassinadri (2024). Rather, the claim that we are committed to is that cognitive processes are partially dependent, and causally influenced by, upon processes outside of the body which are part of the environment the body is situated in and one’s (dispositional) ability to intearct with one’s environment – see Newen et al. (2018)

8 For a similar worry about excessive cognitive offloading being a barrier to the development of intellectual virtues see (Turner, 2022).

9 Note that as a pedagogical approach, UFCT aims to equip students with these skills; we return to this in more detail in Sect. 3.2.

10 We leave open the possibility that one way students might offload inappropriately is by offloading too little, or even by failing to offload, in situations in which it would be beneficial for them to do so. However, in what follows, we focus on *excessive* cognitive offloading to LLMs, given that we’re addressing the worry expressed by commentators that students’ overuse of LLMs will lead to cognitive deskilling.

11 That students are more likely to offload excessively if they are not taught the skills and abilities of delegative intellectual labour is not an evaluation pertaining to their motivation or enthusiasm, but rather that they would not be atypical in being cognitive misers (viz. possess a bias against expending cognitive effort, so offloading appears attractive: see Risko & Gilbert (2016).

12 Many thanks to an anonymous reviewer for discussion on this point.

13 For more on intellectual ethics and its relation to virtue epistemology see (Sosa, 2015, 2021).

14 Carter (2024, p.142–145) includes a third disposition, namely a disposition to reliably assess whether a decision to delegate undermines one’s capacity to direct one’s intellectual life, that is, one’s intellectual autonomy. However, for the purposes of brevity we are concerned only with *sensitivity* and *reliable placement*.

15 Given that virtues are thought to be stable, robust, and successful dispositions (either innate or acquired excellences of character) to act in some specified way, our approach does not succeed in developing virtue nor does it purport to do so. One such reason is that essential to the development of virtue, including *sensitivity* and *reliable placement*, is also the development of specific meta-cognitive emotions such as curiosity and anxiety. Having emotions of this kind can be considered as partially constitutive of the motivational component of some intellectual virtues or play a constitutive role in opening and closing inquiries see: Zagzebski (1996, p.131–132) and Brady (2018). Furthermore, the development of certain meta-cognitive emotions to regulate these emotions would be required for such emotions to be appropriately fitting see: Kurth (2018, 2024); that is for them to be properly considered as partially constitutive of virtue. Many thanks to an anonymous reviewer for discussion on this point. For a critique of the notion that the development of virtue is an achievable aim of education see Kotzee et al. (2021).

16 It has also been proposed that engaging *Memento* can help students learn about ‘The Extended Mind’,see Quinn (forthcoming).

17 Extensive studies have been published in recent years indicating various student responses to AI use in education – see: Chan and Hu (2023); Okulich-Kazarin et al. (2024); Johnston et al., (2024).

18 See also Turner (2002).

19 This upshot is not exclusive to our approach but builds on the potential of engaging with film in educational settings to learn about philosophy. For other examples, see D’Olimpio, L. (2019), MacAllister (2023) and Wartenberg (2007).
